# The Emerging Role of Ferroptosis in Cardiovascular Diseases

**DOI:** 10.3389/fphar.2022.822083

**Published:** 2022-01-26

**Authors:** Min Hong, Jiabing Rong, Xinran Tao, Yinchuan Xu

**Affiliations:** ^1^ School of Medicine, Zhejiang University, Hangzhou, China; ^2^ Department of Cardiology, First Affiliated Hospital, School of Medicine, Zhejiang University, Hangzhou, China; ^3^ Department of Cardiology, Second Affiliated Hospital, School of Medicine, Zhejiang University, Hangzhou, China

**Keywords:** ferroptosis, iron metabolism, lipid peroxidation, GPX4, cardiovascular diseases

## Abstract

Ferroptosis is one type of programmed cell death discovered in recent years, which is characterized by iron-dependent lipid peroxidation and participating in iron, lipid and antioxidant metabolism. Ferroptosis is different from the traditional cell death types such as apoptosis, necroptosis and autophagy in morphology, biochemistry and genetics. Cardiovascular diseases are considered as an important cause of death from non-communicable diseases in the global population and poses a serious threat to human health. Apoptosis has long been thought to be the major type of cardiomyocyte death, but now ferroptosis has been shown to play a major role in cardiovascular diseases as well. This review will discuss related issues such as the mechanisms of ferroptosis and its effects on the occurrence and development of cardiovascular diseases, aiming to provide a novel target for the prevention and treatment of cardiovascular diseases.

## Introduction

Ferroptosis is a regulated death type characterized by lipid peroxidation caused by the accumulation of iron-dependent reactive oxygen species (ROS) in the cell (Dixon et al., 2012). The occurrence of ferroptosis involves multiple pathophysiological processes, and also participates in the occurrence and development of many diseases. Based on the data from World Health Organization (WHO), 17.6 million people worldwide died of cardiovascular diseases (CVDs) in 2012, accounting for 31.4% of global deaths (McAloon et al., 2016). CVDs have become the single most important cause of death from non-communicable diseases in the world (McAloon et al., 2016). Therefore, the investigations on the pathogenesis of CVDs and their therapeutic targets are urgently needed. Current studies have found that ferroptosis is involved in the development and progression of CVDs. This review will discuss the mechanisms of ferroptosis involving iron metabolism disorder and lipid peroxidation induced by oxidative stress, and summarize the research progress of ferroptosis in CVDs, which may provide a potential therapeutic target for the prevention and treatment of CVDs.

## Ferroptosis

Ferroptosis, a phenomenon of non-apoptotic cell death, was first identified by Sonam in 2003 (Dolma et al., 2003), and first named by Dixon in 2012 (Dixon et al., 2012). Dixon named an iron-dependent cell death that can be triggered by the oncogenic RAS-selective lethal small molecule erastin and can be inhibited by the specific inhibitor ferrostatin-1 (Fer-1) as ferroptosis (Dixon et al., 2012). Ferroptosis has its own morphological, biochemical, and genetic characteristics (Dixon et al., 2012). Morphologically, ferroptosis is characterized by mitochondrial shrinkage, with decreased mitochondrial cristae and increased membrane density; Biochemically, the activation of ferroptosis involves the formation of iron-dependent ROS, and this process cannot be inhibited by apoptosis, necrosis or autophagy inhibitors, but can be inhibited by antioxidants and iron chelators; Genetically, multiple genes related to apoptotic or non-apoptotic death are not necessary for ferroptosis, indicating the existence of a unique genetic network for ferroptosis (Dixon et al., 2012). Collectively, these evidences revealed that ferroptosis is a new type of cell death. (Table 1) summarizes the differences between ferroptosis and several other types of regulated cell death.

**TABLE 1:** The differences between ferroptosis and several other types of regulated cell death.

RCD	Ferroptosis	Apoptosis	Necroptosis	Pyroptosis
Characteristic	Lipid peroxidation	MOMP	RIPK and MLKL	GSDMD
Morphological features	Mitochondrial shrinkage, decreased mitochondrial cristae and increased membrane density, increased rupture of mitochondrial membrane	Cell shrinkage, nuclear fragmentation, chromatin margination, membrane blebbing, apoptotic body formation	Swelling of cells and organelles, rupture of plasma membrane, moderate chromatin condensation	Cell swelling and the formation of large bubbles from the plasma membrane, karyopyknosis
Biochemical features	Iron-dependent ROS	Activation of caspases	Activation of RIPK1, RIPK3, and MLKL, proinflammatory Response	Inflammatory cytokines release and proinflammatory response
Triggers	ROS	Micro-environmental disturbance	Ca2+ and ROS overload	PAMPs or DAMPs
Regulatory pathway	System xc- and GPX4, HSPB1-TfR1/FTH1, NF-kB/IL-6/STAT3/hepcidin, ATG5-ATG7-NCOA4 pathway, Keap1-Nrf2 pathway, ATF3-SCL7A11 pathway, SIRT1/p53/SCL7A11 pathway, FSP1-COQ10-NAD(P)H pathway	DR pathway, mitochondrial pathway, endoplasmic reticulum pathway, SMAC/DIABLO/IAP/procaspase-9, HtrA2/IAP/procaspase-9	DR-RIPK1/RIPK3-MLKL related signaling pathways, TLR-RIPK3-MLKL related signaling pathways, ZBP1/RIPK3-MLKL, PKC-MAPK-AP-1-mediated signaling pathways, RIPK3-CaMK II, RIPK3 endoplasmic reticulum stress	Caspase-1 signaling pathways, NLRP3 signaling pathways, LPS-caspase11 signaling pathways
References	1,4-10	11–15	16–18	19,20

AbbreviationRCD, regulated cell death; HSPB1, heat shock protein family B member 1; TfR1, transferrin receptor 1; FTH1, ferritin heavy chain; FTL, ferritin light chain; NF-kB, nuclear factor-kappaB; IL-6, interleukin-6; STAT3, transcription 3; ATG, autophagy-related gene; NCOA4, nuclear receptor coactivator 4; Keap1, kelch-like ECH-associated protein 1; Nrf2, nuclear factor erythroid 2-related factor 2; ATF3, activating transcription factor 3; SLC7A11, solute carrier family 7 member 11; SIRT1, silent information regulator factor 2-related enzyme 1; FSP1, ferroptosis suppressor protein 1; CoQ10, coenzyme Q10; MOMP, mitochondrial outer membrane permeabilization; DR, death receptor; SMAC, second mitochondria-derived activator of caspases; DIABLO, direct IAP binding protein with low pI; IAP, inhibitor of apoptosis protein; HtrA2, high-temperature-requirement A2; RIPK, receptor interaction protein kinase; MLKL, mixed lineage kinase domain-like protein; TLR, toll-like receptor; ZBP1, Z-nucleic acid binding protein 1; PKC, protein kinase C; MAPK, mitogen-activated protein kinases; AP-1, activator protein 1; CaMK II, calmodulin-dependent protein kinase type 2; GSDMD, gasdermin D; PAMPs, pathogen-associated molecular patterns; DAMPS, damage-associated molecular patterns; NLRP3, NOD-like receptor family pyrin domain containing 3; LPS, lipopolysaccharide.

## The Mechanisms of Ferroptosis

**FIGURE 1:**
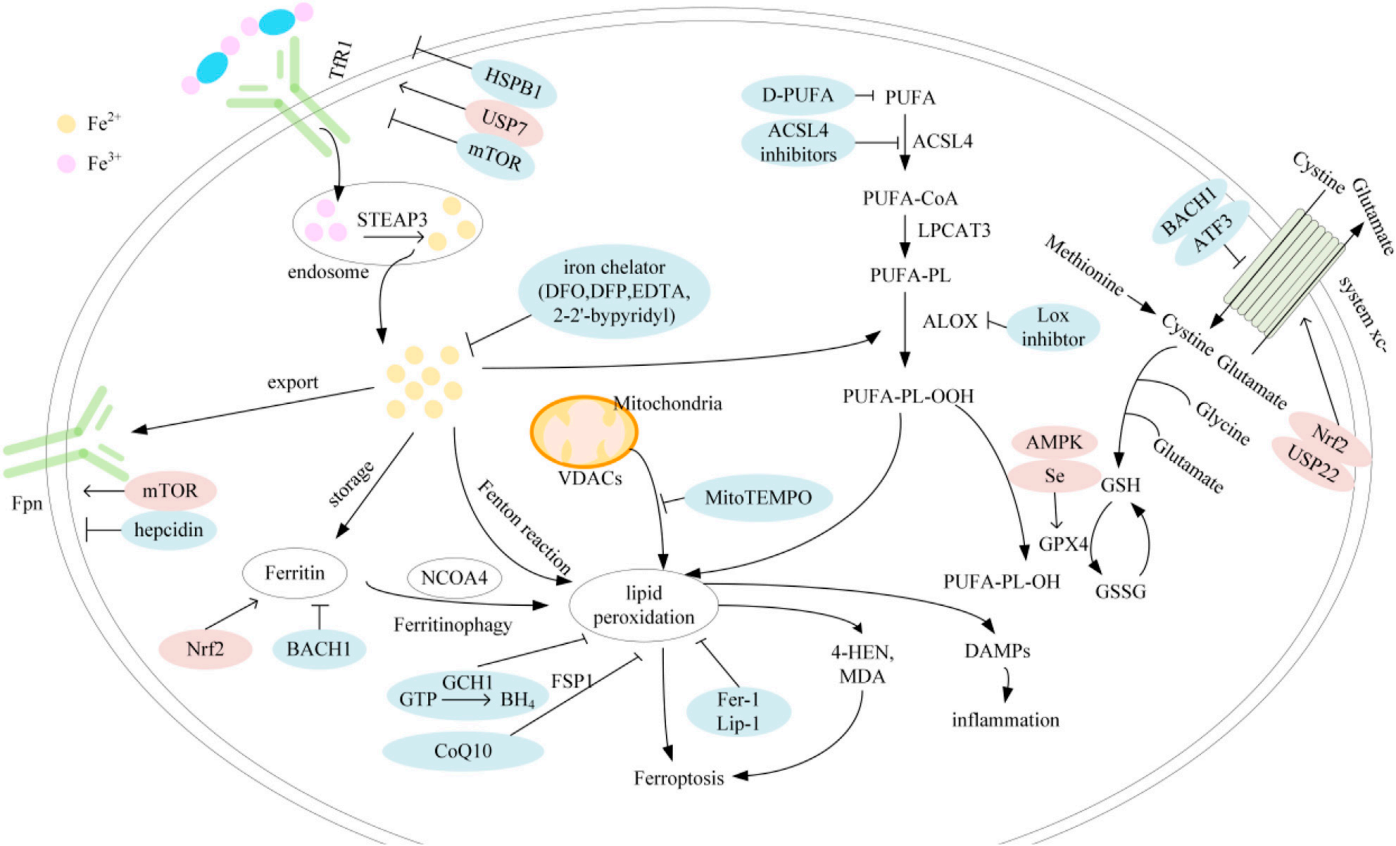
Mechanisms of ferroptosis; Abbreviation: TfR1, transferrin receptor 1; STEAP3, six-transmembrane epithelial antigen of prostate 3; Fpn, ferroprtin; HSPB1, heat shock protein family B member 1; USP7, ubiquitin specific peptidase 7; mTOR, mechanistic target of rapamycin; Nrf2, nuclear factor erythroid 2-related factor 2; BACH1, BTB and CNC homology 1; DFO, deferoxamin; DFP, deferipron; EDTA, ethylenediaminetetraacetic acid; GCH1, GTP cyclohydrolase-1; CoQ10, coenzyme Q10; BH4, tetrahydrobiopterin; FSP1, ferroptosis suppressor protein 1; VDAC, voltage-dependent anion channel; PUFA, polyunsaturated fatty acids; D-PUFA, deuterated-PUFA; ACSL4, acyl-CoA synthetase long-chain family member 4; LPCAT3, lysophosphatidylcholine acyltransferase 3; ALOX, arachidonate lipoxygenase; USP22, ubiquitin specific peptidase 22; ATF3, activating transcription factor 3; AMPK, AMP-activated protein kinase; GSH, glutathione; GPX4, glutathione peroxidase 4; GSSG, glutathione disulfide; DAMP, damage-associated molecular patterns.

The regulatory mechanisms of ferroptosis are complex and involve a series of signaling and molecular metabolic pathways (Figure 1). summarizes the major factors involved in iron, lipid metabolism and antioxidant systems as well as their related pathways.

### Iron Metabolism

#### Circular Iron

Iron is extremely important in various biochemical reactions in cells, and it is an indispensable cofactor for enzymes required for oxygen transport, energy metabolism, DNA synthesis and repair (Hirota, 2019). However, Hydroxyl radicals (HO·) generated by Fe^2+^ through Fenton reaction can also cause biological damage to proteins, lipids and DNA (Hirota, 2019). The maintenance of iron homeostasis is vital for cells to perform normal functions. The majority of iron ion within human body are distributed in hemoglobin (Wang and Pantopoulos, 2011), and the remaining are in the form of ferritin, iron-sulfur groups, and the chelatable labile iron pool (LIP) in peripheral tissues (Lakhal-Littleton, 2019). There are two main sources of iron: release of recycled iron from macrophages and dietary absorption in the duodenum (Ganz and Nemeth, 2012). Dietary Fe^3+^ needs to be reduced to Fe^2+^ by reductases such as duodenal cytochrome b (Dcytb) and then transported by a divalent metal transporter 1 (DMT1) on the tip of small intestinal epithelial cells (Anderson et al., 2012; Hirota, 2019). Fe^2+^ in the small intestinal epithelial cells exit cells through iron transmembrane exporter, ferroportin (Fpn), and are rapidly oxidized to Fe^3+^ by multicopper ferroxidases, such as ceruloplasmin (CP) and hephaestin (HEPE), and then combine with transferrin (Tf) to form a Tf-Fe^3+^ complex (Hirota, 2019; Stoyanovsky et al., 2019). The Tf-Fe^3+^ complex in the plasma binds to transferrin receptor 1 (TfR1) on the surface of tissue cells with high affinity and undergoes endocytosis to enter cells (Wang and Pantopoulos, 2011). Endosomal acidification facilitated by a proton pump triggers the release of Fe^3+^ from Tf, and then the transmembrane epidermal antigen of prostate 3 (STEAP3) reduces Fe^3+^ to Fe^2+^, which enters the cytoplasm through DMT1 (Wang and Pantopoulos, 2011). Cytoplasmic iron is partially transported to mitochondria for the synthesis of iron-sulfur clusters (ISCs) and heme (Wang and Pantopoulos, 2011), partially stored in ferritin, which is composed of two subunit types, ferritin heavy chain (FTH) and ferritin light chain (FTL) (Torti and Torti, 2013), and the remaining iron with redox activity is termed as labile plasma iron (LPI), which has the ability to induce the production of ROS and can be readily taken up by cardiomyocytes and hepatocytes to cause oxidative damage (Fleming and Ponka, 2012).

#### The Role of Labile Iron Pool in the Cellular Response to Oxidative Stress

LIP is the source of iron ions from the Fenton reaction (Kruszewski, 2003). Fe^2+^ reacts with hydrogen peroxide (H_2_O_2_) to generate hydroxyl radical (·OH) and Fe^3+^, and Fe^3+^ can be reduced by superoxide and other cellular reducing equivalents, which allows the next turn of Fenton reaction (Kruszewski, 2003). The whole process called Haber-Weiss reaction, which leads to the continuous generation of hydroxyl radicals (Kruszewski, 2003).

#### The Regulation of Iron Metabolism and Ferroptosis

The intake, utilization, storage and output of iron must be frequently monitored to avoid iron-induced cytotoxicity. Yang et al. found that oncogenic-RAS-signaling augmented the level of intracellular LIP by increasing iron uptake via up-regulation of TfR1 (Yang and Stockwell, 2008; Hirota, 2019) and decreasing the capacity of iron storage via down-regulation of FTL and FTH1 (Yang and Stockwell, 2008), which led to increased sensitivity of cells to erastin and RSL-induced ferroptosis. Besides, heat shock protein family B member 1 (HSPB1) has been shown to play a negative role in ferroptosis. Previously, HSPB1 is considered as a negative regulator of iron accumulation in cardiomyocytes (Sun et al., 2015). Sun et al. further proved that the over-expression of HSPB1 reduces the intracellular iron concentration by down-regulating the expression of TfR1 and up-regulating the expression of FTH1, attenuating the ferroptosis induced by erastin (Sun et al., 2015). Similarly, nuclear factor erythroid 2-related factor 2 (Nrf2), a critical regulator of the antioxidant response, had also been identified as a negative regulator of ferroptosis, enhancing the iron storage capacity of cells by up-regulating the expression of FTH1, thereby reducing TCBQ-induced ferroptotic events (Liu et al., 2021). In addition, Fpn-mediated iron efflux from intestinal epithelial cells and macrophages is critical for systemic iron homeostasis, and this process is negatively regulated by hepcidin (Wang and Pantopoulos, 2011; Shi et al., 2021). Yang et al. found that the anti-rheumatoid arthritis (RA) drug Auranofin at a normal dose (5 mg/kg) could up-regulate the expression of hepcidin-coding gene hepcidin antimicrobial peptide (HAMP) gene through the NF-kB/IL-6/STAT3 signaling pathway, reducing the intracellular iron load and the sensitivity of cells to ferroptosis (Yang et al., 2020), indicating that hepcidin can be utilized as a new strategy for regulating ferroptosis.

Recent researches believe that ferroptosis is a mode of autophagic and caspase-independent cell death (Gao et al., 2016). The initiation of ferroptosis triggers the NCOA4-mediated autophagy pathway, known as ferrotinophagy, to maintain labile iron contents in cells, thereby promoting rapid accumulation of cellular ROS and subsequent further ferroptosis (Gao et al., 2016). It is currently known that NCOA4 selectively interacts with a key residue on FTH1 via the C‐terminal domain, and whether there are other related regulatory pathways for ferrotinophagy is not yet clear (Tang et al., 2018). However, it is more important to note that Gao et al. found that autophagy inhibition has an obviously protective effect at the early phase of ferroptosis, which may be attributed to the inhibition of ROS and lipid peroxide production (Gao et al., 2016). The dual effects of autophagy on ferroptosis need further investigation.

Collectively, the enhancement of iron intake, reduction of stable iron, as well as alleviation of iron outflow can ultimately stimulate oxidative damage and lead to ferroptosis.

### Lipid Metabolism

#### Synthesis and Peroxidation of PUFA-PE

Lipidomic studies have shown that polyunsaturated fatty acids (PUFAs) containing bis-allylic hydrogen atoms that can be easily extracted and susceptible to lipid peroxidation, so phosphatidylethanolamines (PEs) containing arachidonic acid (AA) and its derivative product adrenic acid (AdA) are the most critical phospholipids for oxidation (Stockwell et al., 2017). Two key enzymes are involved in the synthesis of PUFA-PE, named acyl-CoA synthetase long-chain family member 4 (ACSL4) and lysophosphatidylcholine acyltransferase 3 (LPCAT3) (Feng and Stockwell, 2018). The generation of PUFA-PE is via two mechanisms, the nonenzymatic reaction pathways iron-catalyzed free-radical chain reaction and enzymatic reaction, most notably lipoxygenases (LOXs)-mediated oxidation reaction to form peroxides (Feng and Stockwell, 2018), respectively.

#### Regulation of Lipid Metabolism Pathways

Previous investigations revealed that loss of products from ACSL4 and LPCAT3 increases the resistance to ferroptosis in cells (Dixon et al., 2015), especially the modulation of ACSL4-dependent phospholipids is a key determinant of sensitivity to ferroptosis (Doll et al., 2017). Meanwhile, 5-hydroxyeicosanetetraenoic acid (5-HETE) generated from excess AA-CoA catalyzed by ACSL4 was also found to be an important mediator of ferroptosis, and Zileuton, a pharmacological inhibitor of 5-HETE, could block ferroptosis in ACSL4-overexpressed cells (Yuan et al., 2016). Doll S et al. found that thiazolidinediones (TZDs), a kind of peroxisome proliferator–activated receptor-γ (PPARγ) agonists, effectively inhibited ferroptosis induced by erastin and prolonged survival time in GPX4−/− mice by selectively inhibiting ACSL4, which is related to the reduction of AA and AdA-containing PE levels triggered by ACSL4 depletion (Doll et al., 2017). These results indicated that ACSL4 can drive ferroptosis by oxidizing membrane phospholipids and is a positive regulator of ferroptosis. Moreover, either genetic deficiency or pharmacological suppression of LOXs was found to prevent ferroptosis induced by erastin (Gaschler and Stockwell, 2017). Wang et al. confirmed that the high level of ROS induced by RSL3 can increase the level of acetyl-CoA and the acetylation of ALOX12 by inhibiting the CSE/H2S system, leading to the occurrence of lipid peroxidation and ferroptosis, and the supplementation of exogenous H2S can reverse RSL3-induced ferroptosis by normalizing oxidative stress, acetyl-CoA content and ALOX12 acetylation (Wang et al., 2021). In addition, LOXs inhibitors, including Baicalein, NDGA, and PD146176, can inhibit RSL3 induced lipid peroxidation and ROS production, thereby avoiding ferroptotic cell death (Probst et al., 2017).

### Antioxidant Metabolism

The antioxidant system can interrupt the lipid peroxidation chain reaction by reducing the lipid peroxides to lipid alcohols, thus becoming the key factor to determine the occurrence of ferroptotic cell death.

#### Glutathione and Glutathione Peroxidase 4

Glutathione (GSH) is the primary antioxidant in the cell, while Glutathione peroxidase 4 (GPX4) is a selenium-dependent enzyme (Yan et al., 2020). Catalyzed by GPX4, GSH can convert toxic lipid peroxides (L-OOH) to non-toxic lipid alcohols (L-OH) (Stockwell et al., 2017). GSH is synthesized by cysteine, glutamate and glycine catalyzed by glutathione synthase (GSS) and glutamate-cysteine ligase (GCL), of which the available cysteine abundance is the key to GSH synthesis (Stockwell et al., 2017). Cysteine has two main sources, one is from the *trans*sulfuration pathway of methionine and the other is from the transformation of exogenous cystine (Stockwell et al., 2017). The cystine/glutamate antiporter system (system xc-) composed of SLC3A2 and SLC7A11 allows exogenous cystine to exchange with glutamate in a 1:1 ratio (Fan et al., 2017), and cystine is finally oxidized to cysteine after entering the cell (Kuang et al., 2020).

Previously, Dixon et al. have shown that in HT-1080 fibrosarcoma cells, RSL3 can directly target GPX4 to inhibit its function, and erastin can directly inhibit the system xc- to reduce the uptake of cystine, and inactivate GPX4 by affecting the synthesis of the necessary cofactor GSH (Yang et al., 2014), leading to alleviate the ability of GPX4 to remove lipid peroxide. It was found that the expression of GPX4 was reduced in the neuronal cells of patients with multiple sclerosis (MS) and the neuronal cells of experimental autoimmune encephalomyelitis (EAE) mice. Besides, the characteristic ferroptosis-like changes in mitochondrial morphology and the evidence of lipid peroxidation including accumulation of MDA and 4-HNE were observed (Hu et al., 2019), suggesting that ferroptosis of neurons caused by GPX4 deficiency may be the main cause of neuronal death in these diseases. Selenium is necessary for the biosynthesis of GPX4 with the active site selenocysteine. When selenium is deficient in cells, the catalytic activity of GPX4 is significantly reduced, increasing the sensitivity of cells to ferroptosis (Alim et al., 2019). Alim et al. found that selenium supplementation can increase the resistance of cells to ferroptosis by driving the transcription of GPX4 (Alim et al., 2019). The disorder of the antioxidant system is mainly related to the specific disorder of its genes, and the transcription of most genes is regulated by redox-sensitive transcription factors. The dysregulation of transcription factors may have a huge impact on the regulation of cellular redox balance. A transcription factor Nrf2 plays an important role in the oxidative defense of cells by regulating its target genes and downstream effects after sensing the oxidative stress environment (Fan et al., 2017). Recently, studies have found that baicalein can simultaneously inhibit GSH depletion and GPX4 degradation by activating the kelch-like ECH-associated protein 1 (Keap1)-Nrf2 pathway to inhibit erastin-induced ferroptosis in PANC1 cells (Xie et al., 2016). In addition, system xc- is one of the key genes regulated by the transcription factor Nrf2. Genetic deficiency of Nrf2 can down-regulate the expression of system xc- through the Nrf2-Keap1 pathway, leading to erastin-induced ROS accumulation in glioma cells and increasing the sensitivity of cells to ferroptosis (Fan et al., 2017). Targeted regulation of Nrf2 paves a new regulatory direction for ferroptosis. In addition, Wang et al. proposed that activating transcription factor 3 (ATF3) can inhibit the expression of SLC7A11 by binding to the promoter of SLC7A11, thereby inhibiting the system xc- and promoting ferroptosis independent of p53 (Wang et al., 2020a).

#### Coenzyme Q10

CoQ10 is a lipophilic electron carrier that participates in the mitochondrial respiratory chain and acts as a lipophilic radical scavenger in the plasma membrane (Santoro, 2020). Bersuker et al. found that ferroptosis suppressor protein 1 (FSP1), a key component of non-mitochondrial CoQ10, was recruited into the plasma membrane through myristoylation of specific N-terminal sequences to capture lipotropic free radicals and exert antioxidant effects (Srinivasula et al., 2001; Wei et al., 2001; Hegde et al., 2002; Lee et al., 2003; Chua et al., 2007; Linkermann et al., 2013; Zhang et al., 2016; Lu et al., 2018; Zhu et al., 2018; Bersuker et al., 2019; Toldo et al., 2019; Wang et al., 2020b). The application of edibenquinone (a hydrophilic equivalent of CoQ10) can inhibit ferroptosis induced by FIN56, while statins can further increase ferroptotic cell death by degrading CoQ10 (Shimada et al., 2016), suggesting that CoQ10 can cooperate with GSH to exert an anti-ferroptosis effect.

#### Tetrahydrobiopterin

BH4 is a natural nutrient and a cofactor in a variety of enzymes and GTP cyclohydrolase-1 (GCH1) is the rate-limiting enzyme in BH4 biosynthesis. Highly expressed GCH1 has been shown to inhibit ferroptotic cell death by increasing the concentration of BH4 to eliminate the accumulation of ROS, which is independent of GPX4 (Kraft et al., 2020).

Collectively, these results indicated that either the traditional antioxidant GSH/GPX4, or the emerging antioxidant FSP1/CoQ10, BH4, has displayed the potential effects of inhibiting ferroptotic cell death.

### Relationship Between Iron Metabolism, Lipid Metabolism, and Antioxidant Systems

In the case of iron overload, Fe^2+^ in LIP can generate highly reactive hydroxyl free radicals through Fenton reaction, which can peroxidize with lipids to produce lipid peroxides, and Fe^3+^ can generate lipid peroxide radicals by reacting with lipid peroxides to form a peroxide chain reaction (Nakamura et al., 2019). Under normal circumstances, toxic lipid peroxides produced by cell metabolism can be reduced by antioxidants to non-toxic lipid alcohols (Stockwell et al., 2017), however, once the generation of lipid peroxides exceeds the ability of antioxidant scavenging, accumulated lipid peroxides can further attack neighboring PUFAs, forming new lipid peroxides and lipid peroxides, ultimately leading to the enhancement of lipid peroxidation. On the one hand, the continuous peroxidation of PUFAs changes the physicochemical properties of cell membranes, leading to the loss of membrane stability and integrity (Hassannia et al., 2020)and the imbalance of ion homeostasis inside and outside the cell (Magtanong et al., 2016). On the other hand, toxic aldehydes such as 4-hydroxynonenal (4-HNE) and malondialdehyde (MDA), the breakdown products of lipid peroxides, can further react with proteins and destroy essential proteins, promoting cell death (Hassannia et al., 2020). In the case that the lipid peroxidation reaction is not terminated, the accumulation of oxidative damage eventually leads to ferroptotic cell death.

### Ferroptosis and Inflammation

Inflammatory response is the immune response to various environmental stimuli inside and outside the cell. As an important pathological process of many diseases, moderate immunity has a protective effect, while excessive immune response is harmful. Multiple evidences have revealed that the inflammatory response is involved in the process of ferroptosis. In a mouse model of oxalate-induced acute kidney injury, neutrophil infiltration and expression of pro-inflammatory cytokines (CXCL-2 and IL-6) were inhibited by Fer-1 (Linkermann et al., 2014); Intracerebral hemorrhage (ICH) is often accompanied by secondary brain injury (SBI) caused by oxidative stress, inflammation and cell death. Fer-1 treatment significantly reduced the levels of inflammatory cytokines IL-1β and TNF-α in cerebrospinal fluid in ICH rat models, suggesting that inhibition of ferroptosis with Fer-1 alleviates inflammation (Zhang et al., 2018). These results suggest that ferroptosis may be a promoter of inflammation.

Macrophages are widely found in all tissues and are crucial for controlling and regulating the local microenvironment homeostasis, playing a central role in inflammation, immune response and host defense. Macrophages can change into several phenotypes with different functions in response to different environmental factors and pathophysiological changes. In response to stimulators such as IFN-γ and LPS, macrophages form an M1 phenotype expressing specific biomarkers such as CD80 and CD86 through classical activation, and secrete pro-inflammatory cytokines including iNOS, IL-6, IL-12 and TNF-α to promote inflammation as well as trigger immune responses. On the contrary, in response to lL-4 and IL-13, macrophages generate M2 phenotype through alternative activation. M2 macrophages do not express iNOS, but up-regulate the expression of ARG1, IL-10, TGF-β and other anti-inflammatory factors, which participate in tissue repair, inhibition of inflammation and immune response (Martinez et al., 2008). A recent study has found the inhibitory role of M1 macrophages in ferroptosis. After the macrophages were treated with RSL3, M1 macrophages showed great resistance to ferroptosis, which was different from M2 macrophages. Inhibiting iNOS expression in M1 macrophages showed increased sensitivity to ferroptosis induced by RSL3, while the enhanced iNOS expression in M2 macrophages increased the resistance to ferroptosis, suggesting that iNOS/NO can regulate the sensitivity of M1 and M2 macrophages to ferroptosis. Further investigations showed that low levels of nitroxygenated PE products were detected in RSL3-treated cells in the presence of NO supplementation in M2 macrophages, suggesting that NO can interact with the enzymatic reaction intermediates or lipid radical intermediates triggered by RSL3 to inhibit the execution of the ferroptosis program (Kapralov et al., 2020). The course of atherosclerosis is accompanied by ferroptosis. Under the condition of iron overload, macrophages are often polarized to M1 (Xia et al., 2021). M1 macrophages have higher resistance to ferroptosis due to their high expression of iNOS/NO. However, the pro-inflammatory effect of M1-polarized macrophages aggravates the inflammatory response and oxidative stress in atherosclerosis. ROS produced by iron overload promotes lipid peroxidation in macrophages, resulting in increased instability of atherosclerotic plates and deteriorating atherosclerosis. Macrophages play an important role in inflammation and injury repair after myocardial infarction. In the early stage of myocardial infarction, macrophages often polarize into M1 type and perform local inflammatory response by releasing various proinflammatory cytokines such as IL-1β, IL-6, TNF-α and iNOS to clear necrotic myocardial cells, while in the late stage of myocardial infarction, M2 macrophages produce IL-10 and ARG1 to inhibit inflammatory response and promote the repair of cardiac tissue (Cheng and Rong, 2018). Shiraishi et al. found that M2 macrophages showed enhanced repair ability after myocardial infarction, and mice with selective M2 macrophage depletion had a significantly increased risk of cardiac rupture (Shiraishi et al., 2016). Li et al. found that the loss of CD226 could promote the transformation of macrophages from M1-phenotype to M2-phenotype, that is, accelerate the transformation from pro-inflammatory macrophages to repair macrophages, promoting the repair of infarcted myocardium and the improvement of cardiac function (Li et al., 2020). Compared with M1 macrophages, M2 macrophages play a more significant role in cardiac repair after myocardial infarction. In the model of myocardial infarction, macrophage polarization is affected by many regulators, such as collapsin response mediator protein-2 (CRMP-2), IRF5, and voltage-gated sodium Channels (VGSC) can induce M1 polarization, while Dendritic cells (DCs), mesenchymal stromal cells (MSCs), T regulatory cells (Tregs), fibroblast growth factor-9 (FGF-9) and colony stimulating factor receptor 1 (CFS-1R) can promote M2 polarization (Cheng and Rong, 2018). Ferroptosis is involved in the occurrence and development of myocardial infarction and promotes M1 polarization of macrophages in infarcted myocardium. Timely ferroptosis intervention to terminate the proinflammatory state of M1 is very important for cardiac repair. In conclusion, regulation of macrophage polarization under different pathophysiological conditions provides new insights into macrophage plasticity in treating ferroptosis-related cardiovascular diseases.

The specific regulatory process by which ferroptosis initiates the inflammatory response remains largely unknown, and one view is that ferroptosis affects inflammation through immunogenicity. Wen et al. have demonstrated that one of the pro-inflammatory features of ferroptosis is autophagy-mediated release of high mobility group box 1 (HMGB1) (Wen et al., 2019). HMGB1 is a nuclear protein that plays an important role in the regulation of events such as DNA transcription and repair. However, when HMGB1 is released by cell injury or death, it is regarded as a damage-associated molecular patterns (DAMPs) that promotes a range of inflammatory responses and amplifies cell death. HMGB1 binds to advanced glycosylation end-products specific receptor (AGER) to release TNF, mediating immune inflammatory response (Wen et al., 2019). Further studies showed that macrophages take up Kras G12D released by ferroptotic cancer cells through the AGRE pathway, which promotes macrophage M2 polarization through STAT3-dependent fatty acid oxidation to produce an anti-inflammatory immune response (Dai et al., 2020a). In addition to the AGRE pathway, the TLR4 pathway is also involved in the inflammatory response induced by ferroptosis. After heart transplantation, DAMPs released from ferroptotic cells stimulates type I IFN production via TLR4/Trif, which initiates neutrophils recruitment to the transplanted heart to produce inflammatory response (Li et al., 2019). In rhabdomyolysis associated acute kidney injury, ferroptosis-related inflammation is induced by the expression of pro-inflammatory cytokines and chemokines such as CCL-2, IL-6, and TNF-α via the TLR4/NF-κB pathway (Guerrero-Hue et al., 2019). DAMPs released by ferroptosis also affect the STING1 pathway, which plays an important role in initiating innate immunity against microbial infections. Ferroptotic cells release oxidized nucleobases (for example, 8-OHG) to promote the migration and activation of macrophages through the activation of STING, accompanied by the production of cytokines such as IL-6 and iNOS and inflammatory response (Dai et al., 2020b). Distinct DAMPs shape the inflammatory response from different pathways.

There is a complex relationship between ferroptosis and arachidonic acid metabolism. The main component of cell membrane lipids is arachidonic acid, which is not only the substrate for lipid peroxidation, but also for the formation of proinflammatory mediators. Arachidonic acid can produce proinflammatory mediators through three pathways: lipoxygenase (LOX) pathway, cyclooxygenase (COX) pathway and cytochrome 450 (CYP450) pathway (Wang et al., 2019). In the LOX pathway, LOX not only mediates the production of enzyme-induced lipid peroxides, but also indirectly promotes ferroptosis by increasing the instability of GPX4 through LOX-derived proinflammatory factor metabolites such as leukotriene, HETE and oxo eicosanoids (Proneth and Conrad, 2019). In the COX pathway, arachidonic acid is catalyzed into various bioactive prostaglandins (such as PGE2, PGD2, PGI2, TXA2, etc.), which play an important role in inflammatory response (Wang et al., 2019). Ferroptosis up-regulates the expression of PTGS2 (the COX2 encoding gene), further promotes the secretion of inflammatory signaling molecules (Chen et al., 2019a), while GPX4 down-regulates the activity of LOX and COX by reducing the production of lipid peroxides (Huang et al., 1999). These results suggest that ferroptosis and inflammation may complement each other. Ferroptosis and inflammation are closely related, but the mechanisms of their interaction and how to strictly regulate ferroptosis to improve the treatment of inflammation still need to be further investigated.

### Ferroptosis and Myocardial Energy Metabolism

Mitochondria account for one third of the volume of cardiomyocytes in the healthy adult heart and reflect the high myocardial oxidative capacity to meet the high energy requirements of myocardial contraction. Under normal oxygen supply, mitochondria provide 95% of adenosine triphosphate (ATP) through oxidative phosphorylation (OXPHOS), with the remaining 5% come from glycolysis and the tricarboxylic acid cycle. β -oxidation of long-chain fatty acids is the main source of cardiac energy by providing the majority (60–90%) of acetyl-CoA, 10–30% of which is provided by glucose and lactic acid, and A small fraction (5–10%) of acetyl-CoA comes from ketones and amino acids (Honka et al., 2021). The heart has the flexibility to change substrate utilization preferences based on substrate availability, oxygen delivery, hormone concentration, and cardiac nutritional status in order to provide the energy needed to maintain normal cardiac pump function. However, this flexibility in substrate selection is limited in the course of cardiac disease. Oxidative stress associated with many cardiovascular diseases can lead to mitochondrial dysfunction (Ballinger, 2005). Subsequently, mitochondrial dysfunction, including the transfer of metabolic substrates and impaired activity of electron transport chains, reduces the efficiency of ATP production and further exacerbates cardiac dysfunction. Long-chain fatty acids are the preferred fuel for myocardial energy production, but in the failing heart, energy metabolism is characterized by decreased fatty acid oxidation, increased glucose uptake and glycolysis, and uncoupling between glycolysis and glucose oxidation (Fillmore et al., 2014), resulting in an “energy deficient” cardiac phenotype. Ferroptotic cell death has been proved to be involved in the development of MI/RI, HF and other diseases, but the specific mechanism of its disease development is not clear at present, the mitochondrial damage caused by ferroptosis and the remodeling of cardiomyocyte energy metabolism is worth considered. In cysteine deprivation induced (CDI)-ferroptosis, it has been shown that transient activation of mitochondrial aerobic respiration stimulates the occurrence of ferroptosis, while cells with depleted mitochondria show increased resistance to ferroptosis (Wang et al., 2020c). Under the iron overload and oxidative stress, mitochondria of cardiomyocytes showed increased ROS levels, mitochondrial swelling, impaired DNA synthesis and respiratory chain components (Sumneang et al., 2020). The dysfunction of mitochondrial oxidative phosphorylation results in limited fatty acid utilization. In addition, PPAR regulates energy metabolism at the gene transcription level, and its subtype PPARα activation affects almost all enzymes involved in fatty acid oxidation, which plays a central role in myocardial energy metabolism. Abnormal expression of PPARγ also leads to disturbances in myocardial energy metabolism (Sarma et al., 2012). Myocardial fatty acid oxidation decreased and glucose utilization increased in patients with severe heart failure, and PPARα expression was significantly reduced in left ventricular tissue biopsied from patients with severe heart failure (Sarma et al., 2012). PPARγ agonists have been shown to inhibit Erastin-induced ferroptosis by selectively inhibiting ACSL4’s involvement in lipid remodeling (Doll et al., 2017), therefore, it remains to be determined whether the occurrence of cell ferroptosis and the reduction of PPAR co-contribute to the progression of HF.

## Ferroptosis and Cardiovascular Diseases

CVDs remains the main cause of death in the Chinese population at present, with the burden of IHD increasing year by year (Zhao et al., 2019). The pathological basis of IHD is atherosclerosis, which can lead to acute myocardial infarction (AMI) when atherosclerotic plaque ruptures. Restoring the blood perfusion of ischemic myocardium is the best treatment for AMI, however, the subsequent generation of large amounts of ROS can cause further damage to the myocardium, which is called ischemia reperfusion injury (I/RI) (Stamenkovic et al., 2019). Cardiomyocyte death is the root cause of HF. Therefore, the prevention of ferroptotic cardiomyocyte death is a new direction to prevent HF. (Table 2) summarizes the role of ferroptosis in cardiovascular diseases.

**TABLE 2:** Ferroptosis involved in cardiovascular disease findings.

Disease types	Animal models	Ferroptosis intervention	Outcomes	Ref
Atherosclerosis	Apolipoprotein E-deficient mice	Overexpression of GPX4	Overexpression of GPX4 inhibits the development of atherosclerosis by decreasing lipid peroxidation and inhibiting the sensitivity of vascular cells to oxidized lipids	Guo et al. (2008)
Myocardial infarction	MI mouse model (through LAD ligation)	—	GPX4 protein levels decreased during the early and middle stages of MI and that downregulation of GPX4 might contribute to ferroptotic cell death of cardiomyocytes during MI.	Park et al. (2019)
Myocardial ischemia/reperfusion injury	IRI mouse model (through brief coronary ligation)	Overexpress of mTOR	Maintaining iron homeostasis through increasing the instability of TfR1 mRNA and upregating the expression of ferroportin protects cardiomyocytes after ischemia-reperfusion injury	Baba et al. (2018)
	IRI mouse model	Overexpress of USP22	USP22 overexpression can reduce the occurrence of lipid peroxidation and inhibit ferroptosis-induced cardiomyocyte death via the SIRT1/p53/SLC7A11 association	Ma et al. (2020)
	H9C2 cells			
	IRI model of isolated perfused mice hearts	Liproxstatin-1 (Lip-1)	Lip-1 can reduce the size of myocardial infarction and maintain the integrity of mitochondrial structure and function by decreasing VDAC1 level and increasing GPX4 level to protect the heart after ischemia-reperfusion	Feng et al. (2019)
	IRI mouse model	Genetic or pharmacological approaches lowering mitochondrial iron at baseline	reduced cardiac damage from I/R	Chang et al. (2016)
Heart failure	Erastin- or isoprenaline (ISO)-treated H9C2 myocytes *in vitro* and in rats with aortic banding inducing HF	Puerarin	Puerarin inhibited the occurrence of ferroptosis by decreasing ROS content and increasing GPX4 expression level, thus playing a cardiac protective role in HF rats, and its inhibition of ferroptosis may be related to the regulation of NOX4 signaling	Liu et al. (2018)
	Murine models of doxorubicin (DOX)-induced cardiomyopathy	DOX	DOX upregulation of Hmox1 through Nrf2/Hmox1 axis leads to myocardial iron overload and ferroptosis, which is inconsistent with previous reports of cardiac protective effect of Hmox1	Fang et al. (2019)
	Murine models of doxorubicin (DOX)-induced cardiomyopathy	DOX	Acot1 may play a protective role in DOX-induced cardiomyopathy through remodeling of free fatty acid composition and subsequent desensitization of cardiomyocytes to ferroptosis	Liu et al. (2020)
	Acot1 knock-down cardiomyocytes			
	Acot1 overexpression cardiomyocytes			
Diastolic dysfunction	Aged rabbit heart	Deferoxamine	Increased LMWI content and lipid peroxidation in aged rabbit hearts lead to iron-dependent cardiac oxidative stress and hemodynamic dysfunction, while deferoxamine could reduce myocardial lipid and protein oxidation and improve cardiac function in aged rabbits	Lapenna et al. (2018)
Sepsis	Cecal ligation and puncture (CLP)-induced septicemic mice	Dexmedetomidine (Dex)	CLP significantly decreased the protein expression levels of GPX4, SOD and GSH, however, Dex plays a cardioprotective role by reducing iron concentration by decreasing HO-1 expression and increasing GPX4 expression to inhibit ferroptosis	Wang et al. (2020d)

### Atherosclerosis

Atherosclerosis is a chronic vascular inflammatory disease caused by oxidative stress and is a major cause of the development and progression of CHD (Rao et al., 2019). The formation of atherosclerotic plaques is closely related to ferroptosis due to iron deposition and lipid peroxidation of vascular endothelial cells (Matthews et al., 1997). Guo et al. proved that GPX4 overexpression can slow down the development of atherosclerosis by inhibiting lipid peroxidation in the arteries in apolipoprotein E-deficient mice (Guo et al., 2008). Meanwhile, the iron-chelating agent deferiprone can improve vascular endothelial function and defer the progression of atherosclerosis in animals (Matthews et al., 1997; Duffy et al., 2001). Ferroptosis promotes the formation and development of atherosclerotic plaques. As previously mentioned, ferroptosis can produce a pro-inflammatory effect through the release of DAMPs. In addition, fatty streak formed by oxidized phospholipids (ox-PLs) accumulated under endothelial cells is an early marker of atherosclerosis, and ox-PLs can also promote macrophages to activate inflammatory response and phagocytose ox-LDL to form foam cells to promote the development of atherosclerosis (Zhong et al., 2019). Iron overload accelerates endothelial cell dysfunction through its pro-oxidation and pro-inflammatory effects, including increasing monocyte and endothelial cell adhesion and promoting the expression of inflammatory factors such as vascular cell adhesion molecule 1 (VCAM1), monocytechemoattractantprotein-1 (MCP-1) and intercellular adhesion molecule-1 (ICAM-1). Iron overload also forms a pro-oxidation microenvironment for foam cell development by affecting vascular smooth muscle function (Zhong et al., 2019). A recent study showed that iron overload also promotes ferroptosis in foam cells and promotes the expression of inflammatory cytokines IL-1β and IL-18, while SIRT1 reduces ferroptosis in foam cells by activating autophagy (Su et al., 2021). SIRT1 may be a therapeutic target for atherosclerosis, but further studies are needed to clarify the complex mechanisms of autophagy and ferroptosis in macrophages in the context of atherosclerosis. In summary, iron overload, oxidative stress, as well as lipid peroxidation in the ferroptotic environment play a crucial role in the process of atherosclerosis.

### Myocardial Infarction and Myocardial Ischemia/Reperfusion

Myocardial infarction (MI) is a common and high-risk cardiovascular disease (Zhu and Sun, 2018). When MI occurs, cardiac ischemia can lead to the death of cardiomyocytes, and the damaged myocardial tissue is replaced by fibrous scar tissue without contractile function, therefore, the loss of cardiomyocytes ultimately leads to heart failure (Park et al., 2019). At present, the main treatment for MI is to restore the blood perfusion of the ischemic myocardium as soon as possible, called reperfusion. However, during the recovery of aerobic metabolism, ischemic myocardium can generate free radicals through oxidation to cause further damage to cardiac tissues, including oxidative stress, inflammation, etc., known as ischemia/reperfusion injury (I/RI), which is a more common type of myocardial injury than MI (Zhang et al., 2019). Quantitative proteomics analysis showed that ischemic stress can induce a decrease in the level of GPX4 protein in H9c2 cardiomyocytes, and induce lipid peroxide accumulation in the early stage, which can be inhibited by Fer-1 but not by zVAD (Park et al., 2019). Clinical studies have shown that residual myocardial iron is a risk factor for left ventricular remodeling after reperfusion (Yan et al., 2020), and it is reported that iron chelation therapy with ethylene diamine tetracetic acid (EDTA) can alleviate adverse outcomes in patients with AMI (Lamas et al., 2013). It is obvious that ferroptosis is involved in the development of MI/RI. A recent study found that no significant changes in ferroptosis markers (iron, GPX4, ACSL4, MDA) were observed in rat myocardial ischemia models, and no significant improvement in myocardial injury was observed after deferoxamine intervention. Differently, in the rat MI/R models, the levels of iron, MDA and ACSL4 increased with the prolongation of reperfusion time, accompanied by the decrease of GPX4 level, and deferoxamine intervention can significantly improve myocardial injury. This experiment revealed that ferroptosis mainly occurs in the phase of myocardial reperfusion rather than ischemia (Tang et al., 2021a). The findings provide evidence for whether ferroptosis interventions are needed in patients with MI, depending on the course of the disease. Baba et al. proved that excess iron and ferroptosis in cardiomyocytes after MI are important causes of cell death, and mechanistic target of rapamycin (mTOR), on the one hand, increases the instability of TfR1 mRNA through the downstream target Tristetraprolin (TTP) effect, and on the other hand, up-regulation the expression of ferroportin, which leads to the net effect of reduced intracellular iron, inhibits ROS production and protects cardiomyocytes after MI (Bayeva et al., 2012; Baba et al., 2018). The specific regulatory mechanism of mTOR/TTP needs further study. In the rat model of I/R, it was found that the expressions of ubiquitin-specific protease 22 (USP22), SIRT1 and SLC7A11 were down-regulated, while the expression of p53 was up-regulated (Ma et al., 2020). Subsequently, through the overexpression of USP22 in H9C2 cardiomyocytes, the reduction of myocardial infarct size and the improvement of ventricular function were observed (Ma et al., 2020). USP22 can play an anti-I/RI role by inhibiting ferroptosis through the SIRT1/p53/SCL7A11 pathway. Conversely, USP7 promotes ferroptosis in MI/R by activating the p53/TfR1 pathway (Tang et al., 2021b). Liproxstatin-1 (Lip-1) is a spiroquinoxalinamine derivative, which reduces the myocardial infarct size by down-regulating the voltage-dependent anion channel (VDAC1) and its oligomerization level in the outer membrane of myocardial mitochondria, and generates post-ischemic cardioprotective effect in the myocardium against I/RI by maintaining the integrity of mitochondrial structure (Feng et al., 2019). Besides, pharmacological or genetic reduction of baseline mitochondrial iron has been found to reduce cardiac damage from I/R (Chang et al., 2016), suggesting that mitochondrial iron is an important contributor to ischemic damage to the heart and a new therapeutic target for IHD. BTB and CNC homology 1 (BACH1), a transcription factor that regulates heme and iron metabolism, promotes ferroptosis by repressing the expression of a series of ferroptosis protective genes, including SCL7A11, HMOX1, GCLM, GCLC, FTH, and FTL (Nishizawa et al., 2020). BACH1 gene regulates ferroptosis through iron metabolism and antioxidant metabolism, which is a promising treatment target for I/RI induced by ferroptosis. Britanin is a drug with anti-inflammatory, antioxidant and anti-tumor effects. Lu et al. found in MI/R rat model that Britanin could participate in M/IE protection by up-regulating GPX4 level through AMPK/GSK3β/Nrf2 signaling axis (Lu et al., 2022). There are multiple regulatory pathways for cardiac ferroptosis, MI/RI may trigger ferroptosis by selectively destroying one or more of these pathways. How to develop targeted ferroptosis inhibitors according to different pathways is worth further investigation.

### Heart Failure

HF is the final stage of cardiovascular disease, and the loss of cardiomyocytes leading to reduced myocardial systolic function is the fundamental cause of HF. Thus, targeting cell death mechanisms is a potential therapeutic approach to alleviate and/or reverse HF. Puerarin, which has been proved to improve HF in clinical practice, can inhibit iron overload and lipid peroxidation by upregulating the expression of FTH1 and GPX4, effectively inhibit ferroptotic cardiomyocytes death and improve ventricular function in rats with HF (Liu et al., 2018). DOX-treated mice up-regulate the expression of heme oxygenase-1 (Hmox-1), leading to heme degradation in heart and release of free iron, which accumulates in mitochondria rapidly and causes myocardial damage (Fang et al., 2019), while Fer-1 and Lip-1 can reduce the cardiotoxicity induced by doxorubicin (DOX) (Fang et al., 2019; Koleini et al., 2019), suggesting that ferroptosis plays an important role in DOX-induced HF. DOX can also induce lipid peroxidation through DOX-Fe^2+^ complexes in mitochondria, causing toxicity to the heart, and chelating agents that target Fe^2+^ instead of Fe^3+^ can effectively prevent DOX-induced ferroptosis (Tadokoro et al., 2020), which suggests that mitochondrial-dependent ferroptosis plays an important role in DOX-induced cardiotoxicity (DIC). In addition, acyl-CoA thioesterase 1 (Acot1) catalyzes the decomposition of fatty acyl-CoA into CoA-SH and free fatty acids, remodeling the lipid composition of cardiomyocytes and desensitizing cardiomyocytes to ferroptosis caused by DOX (Liu et al., 2020). Chen et al. found that TLR4 or NOX4 knockdown in rats significantly improved left ventricular remodeling and reduced myocardial cell death in HF rats induced by aortic banding, confirming that toll-like receptor 4 (TLR4)/NADPH oxidase4 (NOX4) participates in regulating autophagy and ferroptosis-mediated myocardial cell death (Chen et al., 2019b). Ferroptosis is involved in the development of HF, but the detailed molecular mechanism of ferroptosis in cardiomyocytes and the regulated signaling pathway need to be further studied.

### Others

According to a meta-analysis of heavy beta thalassemia major in patients with a high incidence of cardiomyopathy, cardiac arrhythmia and HF are the leading causes of death in these patients (Koohi et al., 2019). Because these patients need to rely on regular blood transfusions to inhibit bone marrow activity, which leads to rapid and large amounts of iron accumulation in the body causing oxidative stress and damages cardiomyocytes (Berdoukas et al., 2015). The use of iron chelators reduced mitigate cardiac mitochondrial dysfunction and rapidly ameliorate cardiac function in iron-overloaded animals (Sumneang et al., 2020). Lapenna et al. found that elderly rabbits had higher levels of low molecular weight iron (LMWI) and lipid oxidation in their hearts compared to adult rabbits, and that associated with diastolic dysfunction, while the use of deferoxamine improves cardiac (Lapenna et al., 2018), suggesting that ferroptosis may be involved in the occurrence of cardiac diastolic dysfunction in the elderly rabbits. Targeted interventions in ferroptosis may improve HF due to diastolic dysfunction in elderly patients. Cardiac dysfunction caused by sepsis is accompanied by significant morbidity and mortality. Wang et al. found that myocardial iron concentration increased while GPX4 and GSH protein levels decreased during sepsis. Dexmedetomidine, a α2-adrenergic receptor (α2-AR) agonist, reduces myocardial iron concentration by reducing sepsis induced Hmox-1 overexpression, decreases inflammatory cytokines IL6 and MCP-1 expression, and increased GPX4 activity to reduce cardiac injury (Wang et al., 2020d). These suggest that septic cardiac dysfunction is associated with ferroptosis and provides new insights into the protective mechanism of dexmedetomidine.

## Interventions That Target Ferroptosis to Prevent Cardiovascular Diseases

Ferroptosis related to intracellular iron overload and lipid peroxidation has an important impact on the occurrence and development of CVDs. Targeting ferroptosis is an effective strategy for the treatment of ferroptosis-related CVDs. Ferroptosis can be intercepted by iron chelation therapy and preventing or reversing the occurrence of lipid peroxidation.

### Iron Chelation Therapy

Deferoxamine (DFO), deferiprone (DFP) and deferasirox (DFX) are iron-chelating agents commonly used to eliminate cardiac iron overload, and have been shown to improve heart failure due to cardiac iron overload in adults with severe thalassemia (Cassinerio et al., 2012). They inhibit oxidative stress and ROS production by chelating the accumulated iron in cells. DFO has a strong affinity for iron and has low permeability in cells. Whether it is in MI/R animal models or patients undergoing coronary artery bypass grafting, we have witnessed the effect of DFO in improving heart function (Chang et al., 2016). Besides, a study showed that the combination therapy of two iron chelating agents or the combination therapy of an iron chelating agent and an antioxidant is highly effective in improving cardiac dysfunction of cardiomyopathy (Wongjaikam et al., 2015), but more model studies are still needed in the future to explore its clinical applicability. 2-2′-bipyridyl, another metal chelator with higher cell permeability than DFO, has been shown to reduce iron accumulation, oxidative stress and inflammation in neuronal cells after cerebral ischemia in mice, and improve neurological function (Wu et al., 2012), but its role in MI/R has not been further verified. EDTA is a chelating agent that binds to metal ions such as calcium, lead, iron, and copper. It was shown in a randomized controlled trial (RCT) to reduce the risk of adverse cardiovascular outcomes in MI/R patients (Lamas et al., 2013). Iron chelators can improve the outcome of ferroptosis-related CVDs, but more research evidence are needed to support the routine application of iron chelators in MI and cardiomyopathy.

### Prevent or Reverse Lipid Peroxidation

For lipid peroxidation events, on the one hand, it can be suppressed by reducing lipid peroxidation substrates or inhibiting lipid peroxidase; on the other hand, it can be reversed by enhancing the antioxidant activities of GSH/GPX4 or other classical antioxidants. Phospholipids containing PUFAs become lipid substrates for peroxidation because of their easy extraction of hydrogen atoms. Replacing hydrogen atoms in diallyl with deuterium has been proved to block the peroxidation of lipids (Yang et al., 2016). TZDs can selectively inhibit ACSL4 and the formation of lipid peroxides to inhibit ferroptosis, which has a cardioprotective effect of reducing infarct size in MI/R rats (Doll et al., 2017), but also accompanied by adverse effects of arrhythmia (Palee et al., 2013). The double-sided effects of TZDs on cardiac function limit its clinical application as an inhibitor of ferroptosis. 12/15-LOX has been associated with the development of diseases such as atherosclerosis and HF due to its pro-oxidation and pro-inflammatory effects (Kayama et al., 2009). Baicalein improves cardiac function by specifically inhibiting 12/15-LOX and reducing oxidative stress and inflammation (Ai et al., 2017). Baicalein can also up-regulate GPX4 by activating the Keap1-Nrf2 pathway to produce antioxidant effects (Xie et al., 2016). Fer-1 and Lip-1 are radical-trapping antioxidants (RTAs) that reduce MI/RI by trapping peroxyl radicals (Zilka et al., 2017). MitoTEMPO is a superoxide scavenger targeting mitochondria that significantly reduces MI/R related cardiac dysfunction and mitochondrial damage by removing lipid peroxides in mitochondria (Fang et al., 2019). In addition, Vitamin E, CoQ10 and Omega-3 polyunsaturated fatty acids have all been shown to reduce the incidence of adverse cardiovascular events through their anti-inflammatory and antioxidant effects (Hinman et al., 2018; Bersuker et al., 2019; Watanabe and Tatsuno, 2020).

## Conclusion

At present, the main mechanisms of ferroptosis are iron overload, lipid peroxidation and antioxidant system dysfunction. However, the research on ferroptosis is still in the initial stage, and there are still many questions to be answered urgently. First, the occurrence of ferroptosis involves the regulation of multiple genes in iron, lipid and antioxidant system metabolism, while the specific regulation pathways of these genes have yet to be explored. Second, the current studies on ferroptosis and cardiovascular diseases only involve the superficial phenomena and results, and the detailed role of ferroptotic cardiomyocytes death in the occurrence and development of cardiovascular diseases has not been thoroughly studied, which is still a challenge for precision medicine of the diseases. Finally, as a new type of cell death, whether ferroptosis is related to other regulatory cell death signaling pathways remains to be further investigated. In conclusion, ferroptosis plays an important role in cardiovascular diseases. It is believed that with the progress of basic research and clinical research, further investigations of the mechanisms and intervention approaches of ferroptosis can provide new therapeutic strategies for cardiovascular diseases.
